# Full‐Arch Implant Rehabilitation Integrating Mandibular Movement Records Using Complete Digital Workflows: A Case Report

**DOI:** 10.1155/crid/9947626

**Published:** 2025-12-12

**Authors:** Khanh Long Nguyen, Quang Khanh Chu, Duc Tien Doan, Thanh Tung Nguyen

**Affiliations:** ^1^ Vietnam Cuba Friendship Hospital, Hanoi, Vietnam; ^2^ iMed Dental Clinic, Hanoi, Vietnam; ^3^ National Hospital of Odonto-Stomatology, Hanoi, Vietnam

**Keywords:** case report, digital workflow, full-arch rehabilitation, implantology, mandibular movement records

## Abstract

Full‐arch implant rehabilitation is a demanding procedure where accuracy, occlusal stability, and function remain major challenges. Digital workflows have improved many steps of treatment, but the use of mandibular movement data in prosthetic design is rarely described. This report presents the rehabilitation of a 69‐year‐old male with terminal dentition using a complete digital approach. Six maxillary and five mandibular implants were placed with a stackable surgical guide. CBCT, intraoral and facial scans, Exocad planning, and the PIC system were combined to ensure precise implant positioning. Mandibular kinematic records from the Zebris JMA Optic system guided occlusal adjustment in both provisional and definitive prostheses. The zirconia‐based restoration provided stable function, good esthetics, and high patient satisfaction, with no complications during follow‐up. This case illustrates how incorporating mandibular movement records into a digital workflow can enhance the predictability and clinical outcomes of full‐arch rehabilitation.

## 1. Introduction

Tooth loss caused by aging, trauma, or disease significantly affects oral health, function, and esthetics. Full‐arch implant rehabilitation is a predictable solution, but it remains complex in both the surgical and prosthetic phases [1]. Conventional techniques, while effective, have limitations in accuracy and adaptability for patient‐specific needs [2, 3]. Advances in digital technologies, including intraoral scanning, facial scanning, virtual planning, and mandibular recording, have transformed implant dentistry, improving accuracy and efficiency [4, 5].

Previous reports have shown that digital workflows enhance preoperative planning, implant positioning, and prosthesis fabrication. However, most studies focus on imaging, guided surgery, or CAD/CAM (computer‐aided design/computer‐aided manufacturing) prosthetics, while the integration of mandibular movement data into prosthetic design has rarely been reported. Occlusal function and mandibular dynamics are critical for implant longevity, yet they are often simplified or overlooked in digital protocols. Incorporating mandibular kinematics offers several advantages: It allows prostheses to be designed in harmony with patient‐specific functional pathways, improves occlusal balance, and reduces the risk of implant overload. This case report describes the rehabilitation of a 69‐year‐old patient with terminal dentition using a complete digital workflow. By integrating mandibular kinematic records with virtual planning, surgical guidance, and digital prosthesis fabrication, this report highlights how functional and esthetic outcomes can be improved.

## 2. Clinical Report

A 69‐year‐old man presented for an implant consultation in iMed Dental Clinic with tooth loss and tooth wear on the lower jaw incisors, which directly affect chewing function and facial esthetics. His medical history was unremarkable, with no medications affecting healing or implant integration, and he had no prior bisphosphonate therapy. Additionally, there were no significant oral health issues reported in his family. Previous dental interventions included a ceramic bridge from #12 to #21 and two restorations on an unknown brand in the lower left molar area.

The patient has a bridge from #12 to #21, has lost all molars in the upper arch, all premolars and molars in the lower arch, except #38, and the other teeth had Stage IV periodontitis [1, 6]. There are also two ceramic crowns (#36 and #37) on an unknown implant brand. The patient′s vertical dimension of occlusion has been lowered due to tooth wear on the lower jaw. The smile line is normal, but the midline is slightly tilted to the right. A 13 × 15 cm CBCT (cone beam computed tomography) scan was taken by the CRANEX 3Dx machine (Soredex, Finland), showing severe bone loss in both the maxilla and mandible (Figure 1C). The overall bone density was around D3, and there was enough bone volume for implant placement. According to the Carames classification, the maxilla is CC4a, and the mandible is CC1 [4].

**Figure 2:**
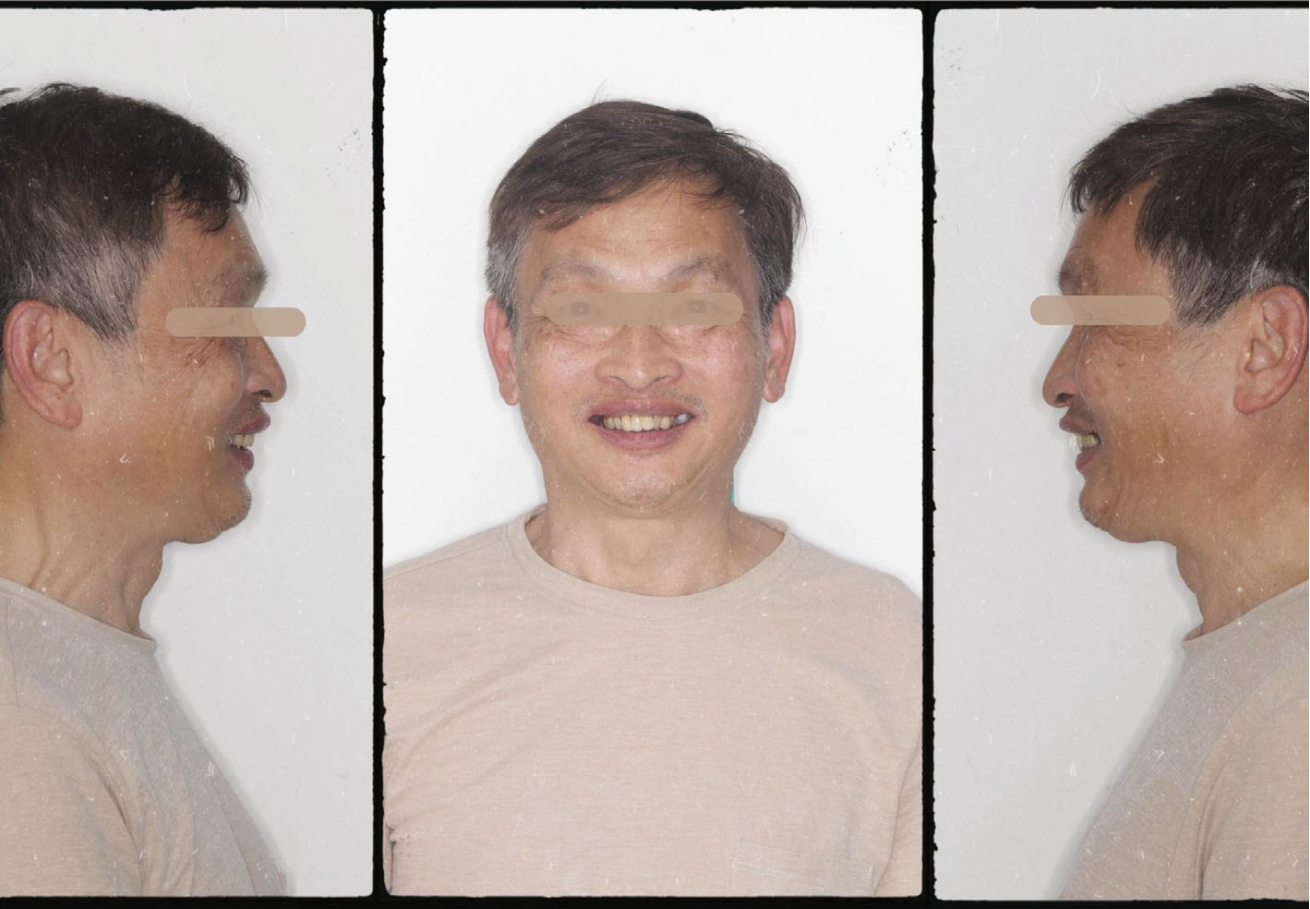
Patient′s extraoral photoset with frontal and lateral views.

**Figure 3:**
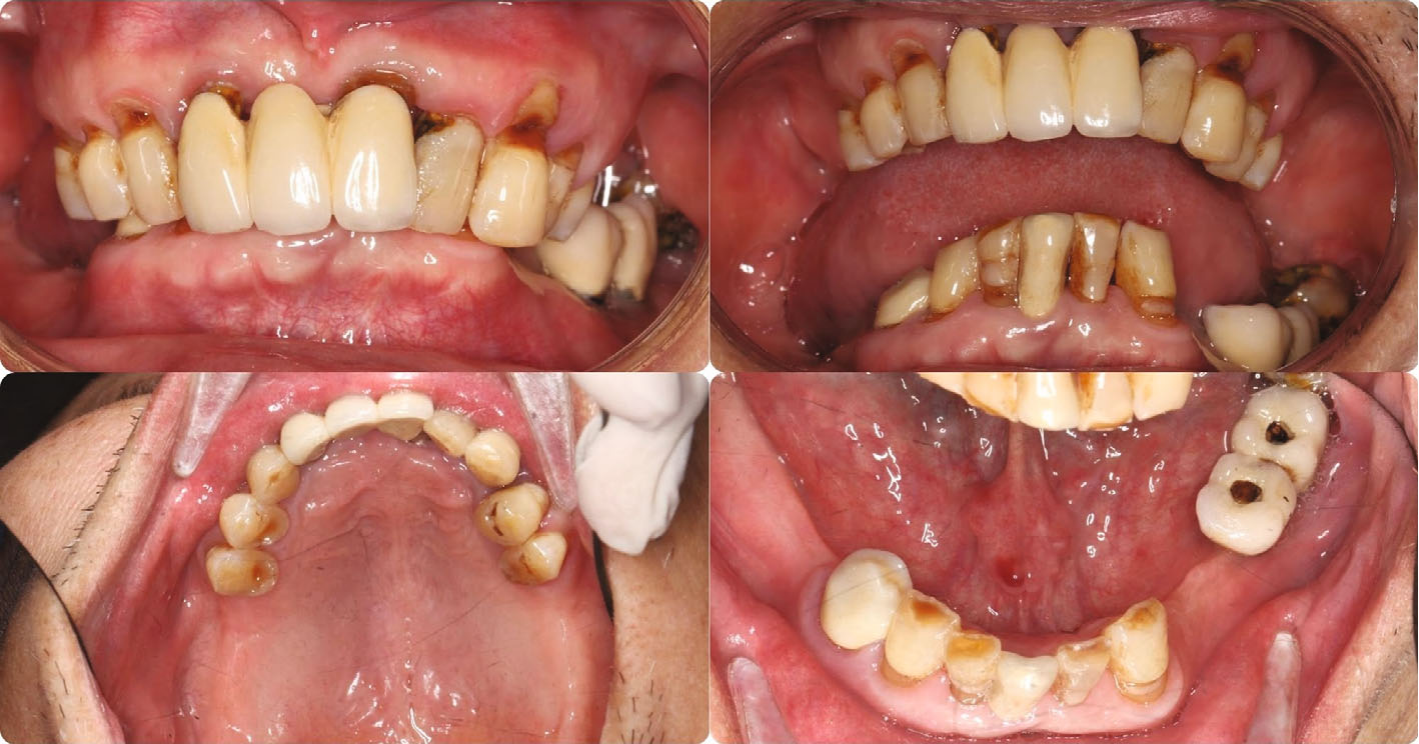
Intraoral photoset.

At the first appointment, we perform all the data acquisition for the patient. The extraoral photo set includes frontal, lateral, and full smile views; the intraoral photo set includes images of both jaws, occlusion, and frontal and lateral views (Figures 2 and 3). All image sets were taken with a Canon RP body and a Canon 100 mm F2.8 lens (Canon Inc., Japan) [5, 7]. The patient′s face was scanned using the Qlone 3D Scanner (EyeCue Vision Technologies LTD, United States) on an iPhone 14 Pro (Apple Inc., United States) (Figure 1a) and exported to the  ^∗^.ply extension [8].

Figure 1(a) Facial scan using Qlone 3D Scanner. (b) Intraoral scan using Trios 3. (c) Panoramic image from the CBCT dataset.

**Figure:**
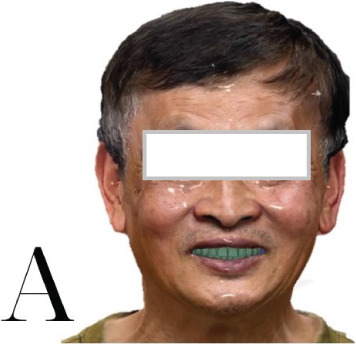
(a)

**Figure:**
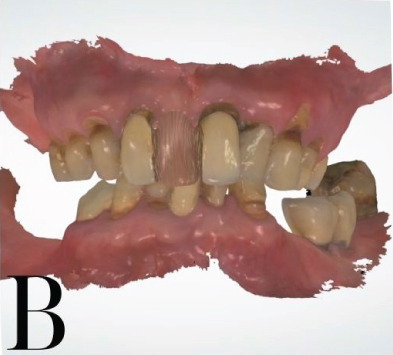
(b)

**Figure:**
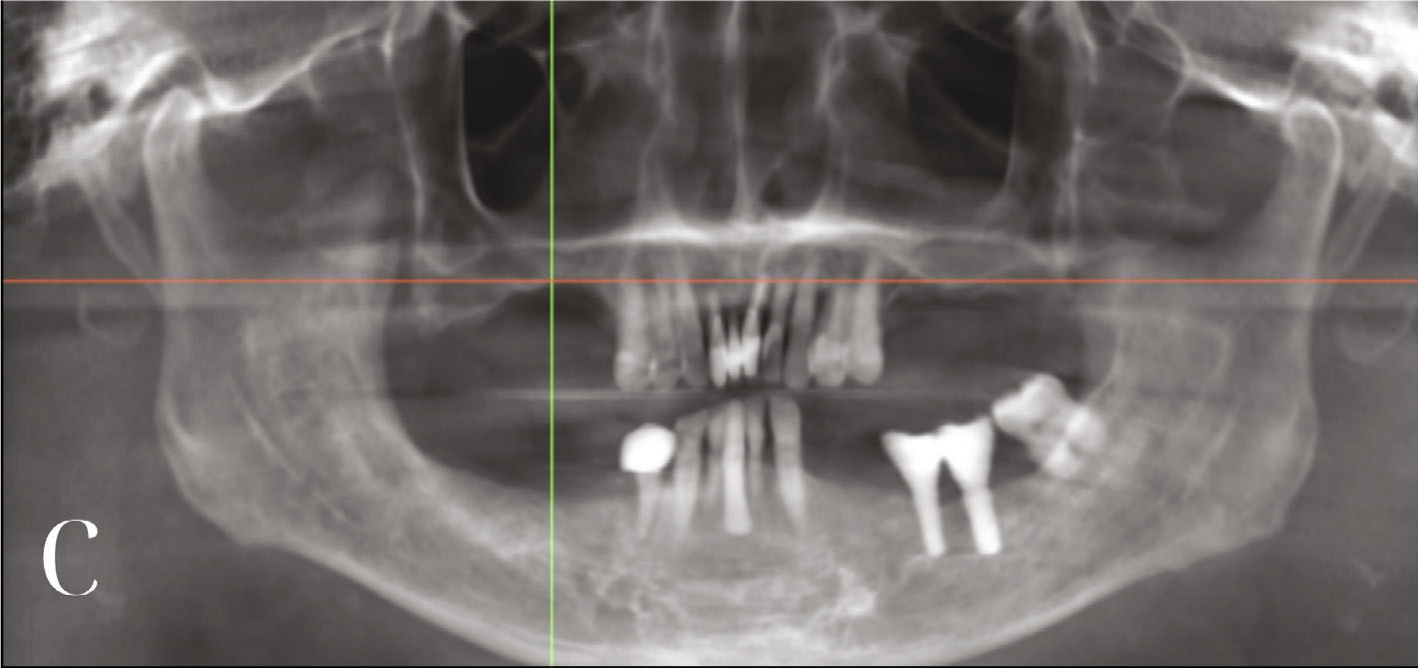
(c)

A leaf gauge was used to increase the patient′s OVD (occlusal vertical dimension) by about 5 mm while maintaining the centric relation [9]. A bite registration material, OBite (DMG Chemisch‐Pharmazeutische Fabrik GmbH, Germany), was used to lock both of the patient′s jaws in this position. An intraoral scanner (Trios 3, 3Shape A/S, Denmark) (Figure 1b) was used to record both jaws, and the data was exported in  ^∗^.dcm format. The patient′s CBCT data was exported in DICOM (Digital Imaging and Communications in Medicine) format for the design of the surgical guide. We used the AI segmentation feature of the BlueSkyPlan software to create STL (stereolithography) versions of the maxilla and mandible from the patient′s DICOM data [10]. This allows us to easily manipulate and fix the coordinates of the 3D files from now until the end of the case.

All the patient′s digital data was combined using Exocad 3.0 (Exocad GmbH, Germany), including the facial scan, intraoral scan, and CBCT STL file. A virtual articulator was embedded to simulate mandibular movement, and the hinge axis was set by both external auditory canals. The condylar angle was set to 42° after examination of the CBCT sagittal slice [11] (Figure 4a). After that, a virtual patient [12] was created with all the information needed for implant planning and prosthetic design. A dual‐arch virtual mockup was created, following Monson′s sphere [13], protrusion, and laterotrusion guidance information on the articulator [12, 14] (Figure 4b).

Figure 4(a) Virtual patient in Exocad software and the method to calculate the condylar angle. (b) Patient′s virtual mockup.

**Figure:**
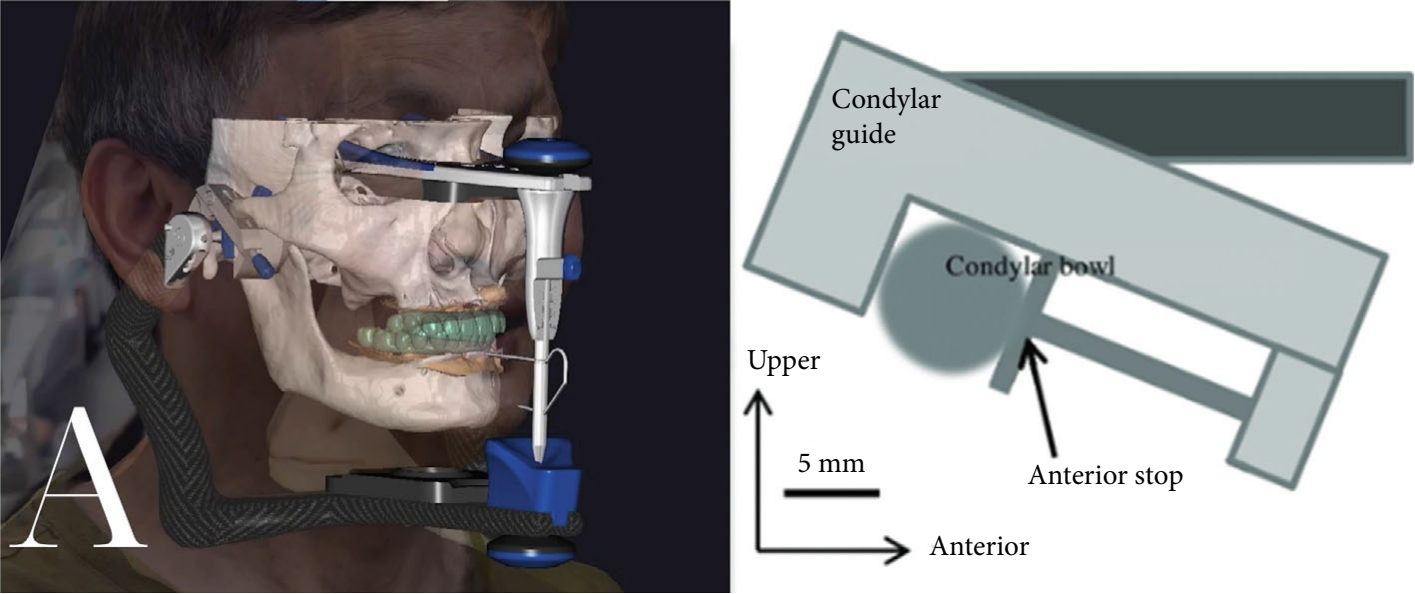
(a)

**Figure:**
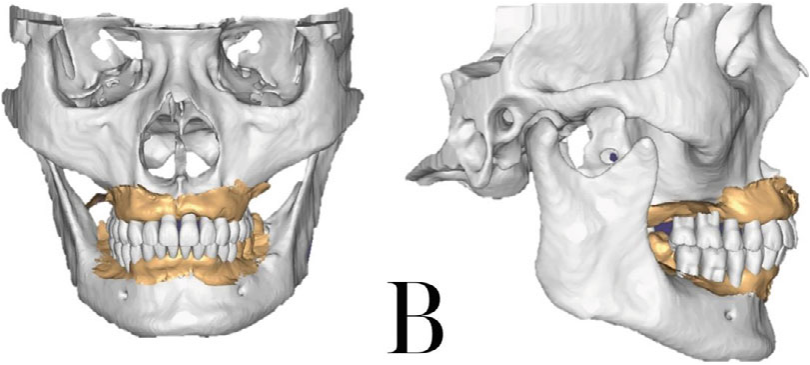
(b)

**Figure 5:**
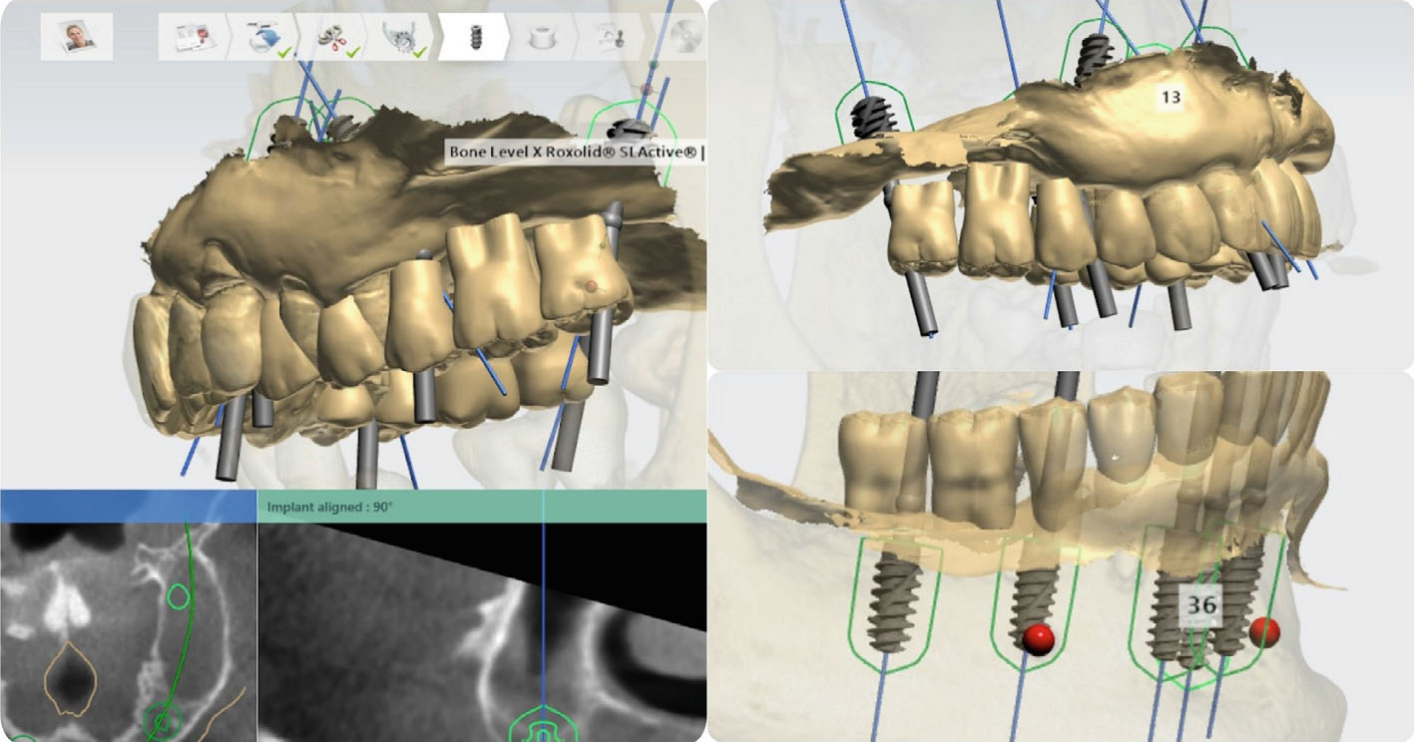
Planning using 3Shape Implant Studio.

**Figure 6:**
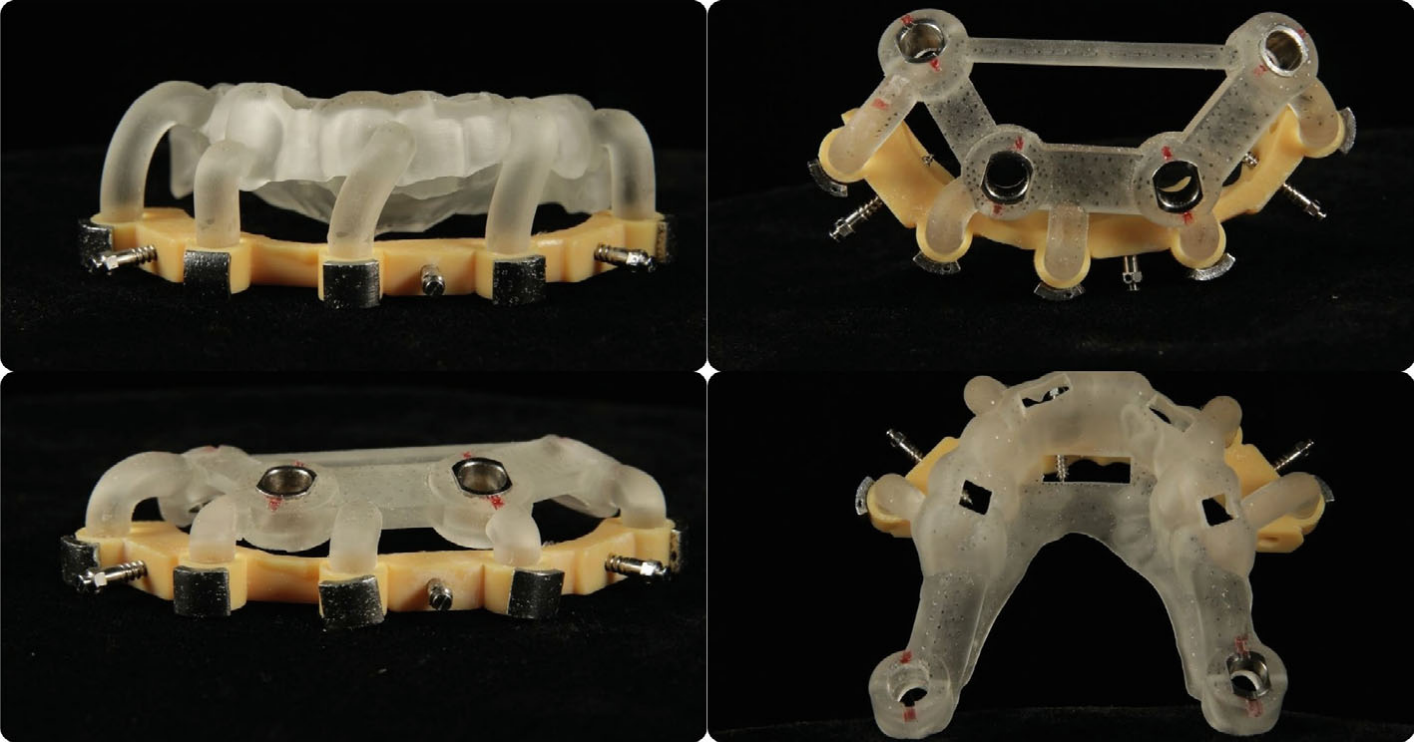
Stackable guide set.

A stackable guide system [15, 16] (Figures 5 and 6) was designed using 3Shape Implant Studio (3Shape A/S, Denmark) and Meshmixer (Autodesk Inc., United States) to place six implants in the maxilla and five implants in the mandible (Bone Level X RB, Straumann AG, Switzerland). Surgical guides were 3D printed with Shining SG01 resin (Shining3D, China).

Implant stability was assessed with the EasyCheck system (Dentium, Korea). Primary stability at placement showed torque values above 35 Ncm, with ISQ (implant stability quotient) readings between 68 and 74.

**Figure 7:**
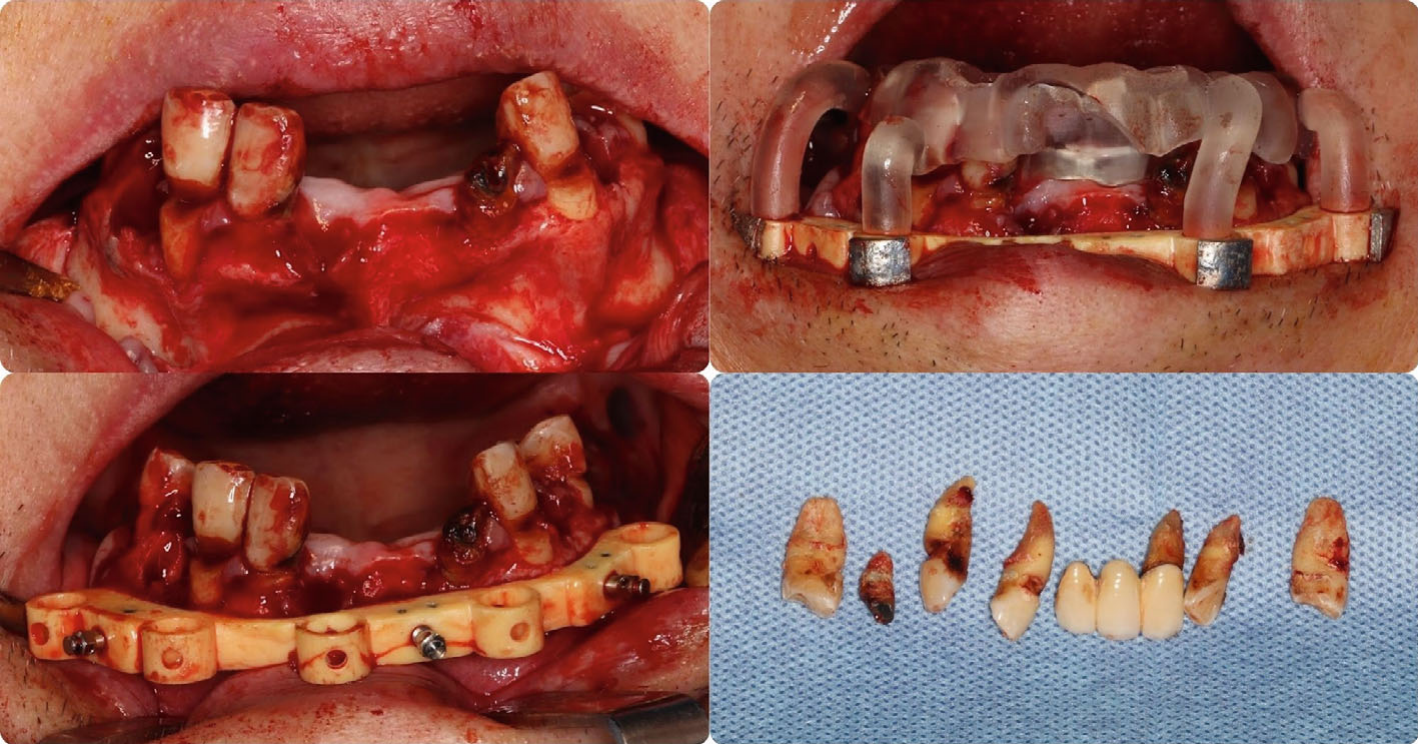
Flap design, tooth extraction, and bone reduction.

**Figure 8:**
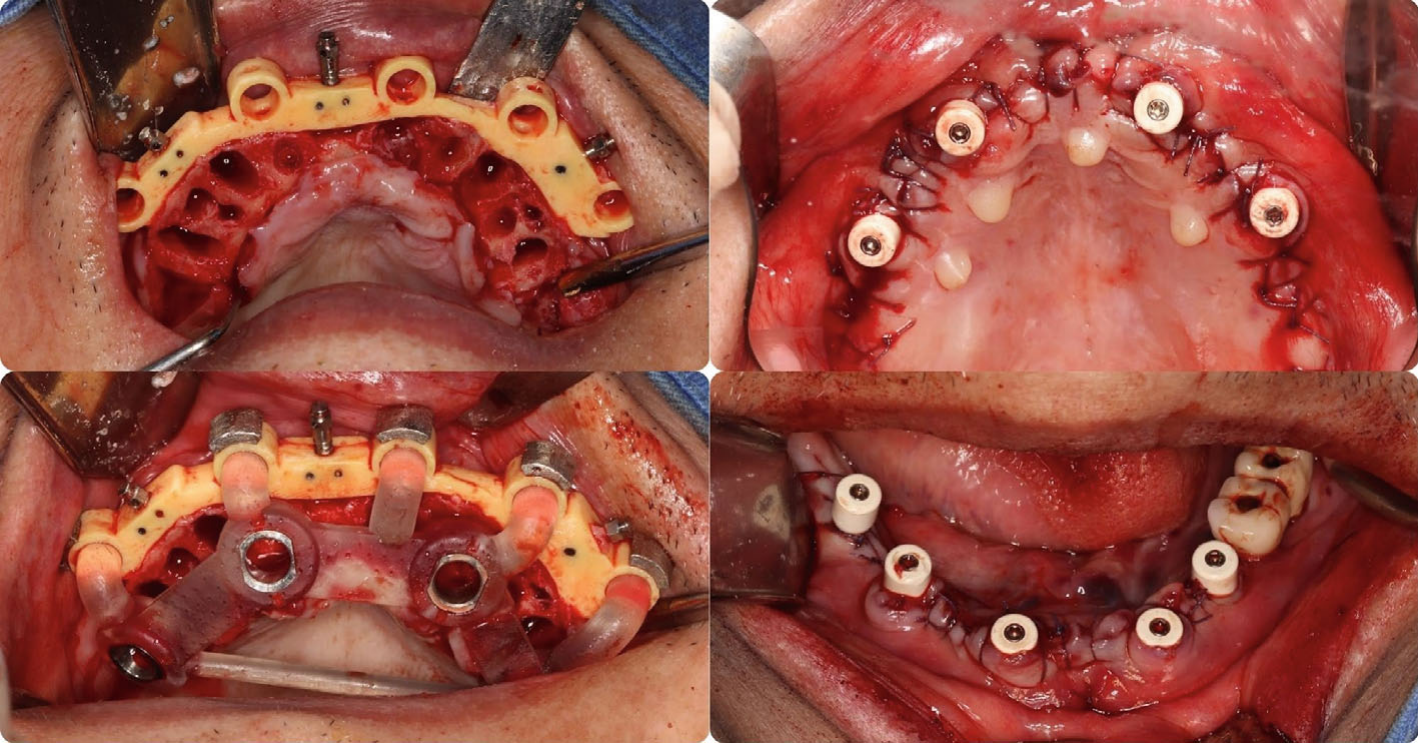
Implant drill and MUA placement.

The patient was put under local anesthesia. A full flap was opened, and all the remaining teeth were extracted. Bone contouring was done according to the surgical guide. Implant placement was completed using Straumann BLX implants, the stackable guide system, and the Straumann VeloDrill System, with multiunit abutments attached to ensure optimal angulation for the prosthetic phase (Figures 7 and 8).

Six composite markers on each jaw were placed for accurate postoperative data alignment. An intraoral scan was performed with all the composite markers placed in the mouth (Figure 8). The implant position was captured using the PIC system (Precise Implants Capture, PIC dental, Spain) to ensure the passive fit of both provisional and definitive prostheses [17, 18] (Figure 9a). A postoperative CBCT scan was performed to confirm the correct position of the implants and capture the implant position for prosthetic design. We continue to use the AI segmentation function of BlueSkyPlan to create STL files of the maxilla and mandible, aligning them to the bone position before surgery in Exocad [19]. This way, we can easily superimpose the soft tissue scan and implant position back to the virtual patient′s coordinates. This approach helps us save time and avoid taking the bite registration after surgery, which could lead to errors due to prolonged mouth opening after the procedure. Two provisionals were designed according to the patient′s virtual mockup, connected through Variobase for Bridge/Bar Cylindrical Coping H 4MM, F. Ø 4.6MM (Straumann AG, Switzerland). The provisionals were milled using the Roland DWX‐52Di Milling Machine (Roland DG Corporation, Japan) with PMMA discs (Aidite Qinhuangdao Technology Co. Ltd., China) and stained with the Optiglaze kit (GC International AG, Japan) [17, 20] (Figure 9b). Two provisionals were seated in the patient′s mouth 1 day after surgery (Figure 9b).

Figure 9(a) Implant position capture with PIC system. F, monolithic PMMA provisional. (b) Provisionals seated after 24 h.

**Figure:**
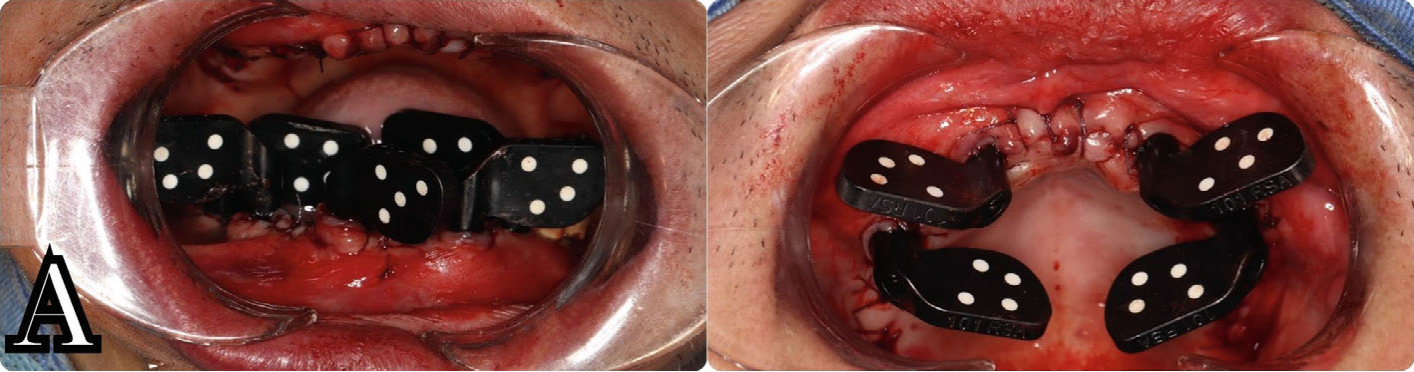
(a)

**Figure:**
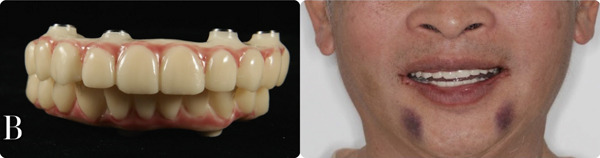
(b)

At 3‐month follow‐up visits, all implants maintained stable ISQ values, confirming continued osseointegration.

After the healing period, the final prosthetic phase was initiated. This phase involved designing and fabricating the definitive prosthesis, which required careful planning to ensure both functional and esthetic excellence.

**Figure 10:**
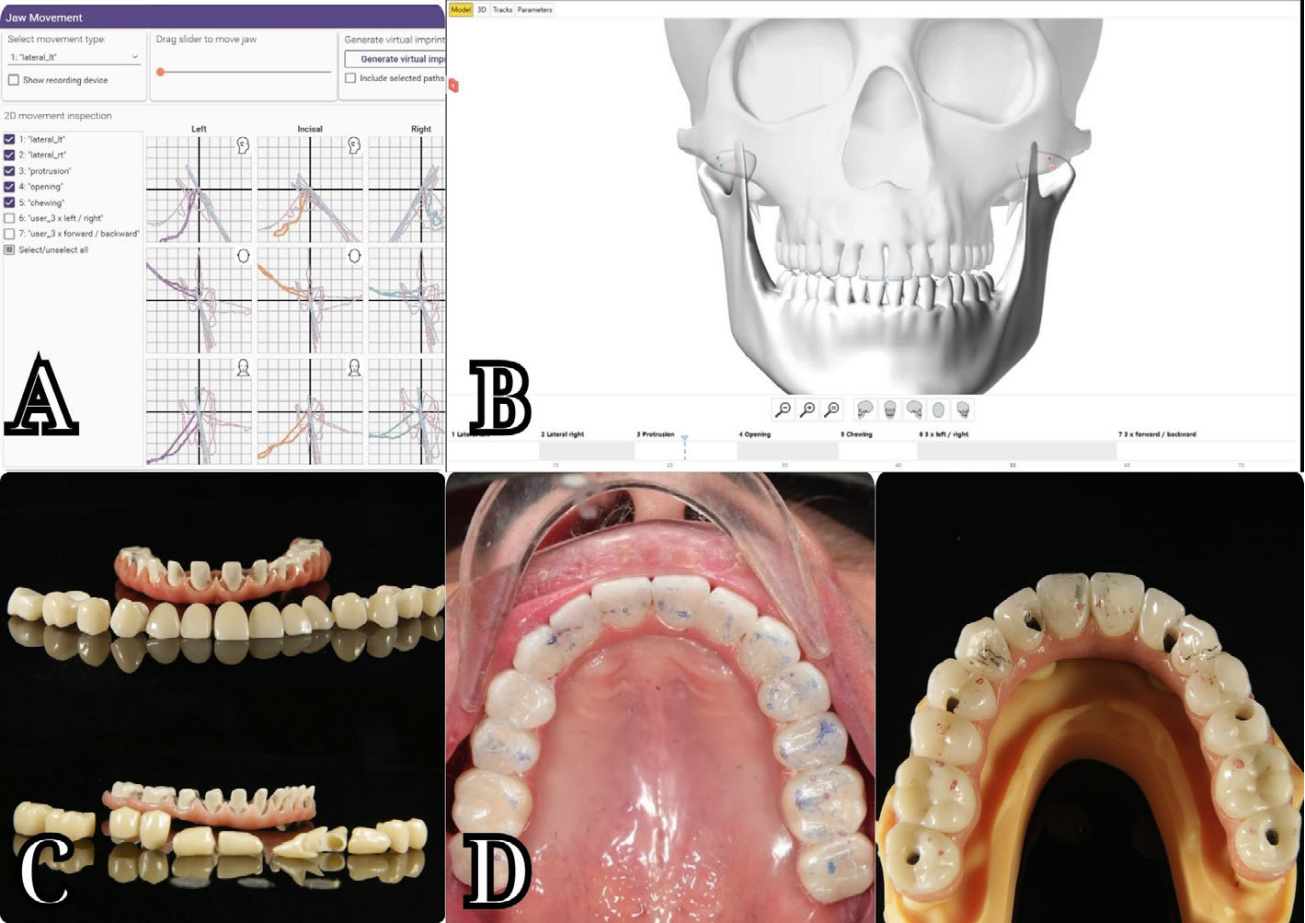
Final prosthesis. (a) Zebris mandibular movement recording was imported into Exocad software to design the final prosthesis. (b) Occlusion analysis inside Zebris software. (c) Titanium frame with composite gingiva and monolithic crown. (d) Patient′s occlusal contact compared with the designed occlusal contact.

To guarantee that the final prosthesis has both esthetic and functional accuracy, the Zebris JMA Optic system [18] (Figure 10a,b)—the mandibular movement recording system—was used in the transitional phase. The patient′s movement data provided by the Zebris system were transferred to Exocad, and a full movement analysis was performed, ensuring that the definitive prosthesis would have a balanced and protected occlusion in protrusion, laterotrusion, and retrusion [21, 22]. The results indicate that the protrusion of the provisional restoration was normal and accurately mimicked the patient′s movements. However, laterotrusion to the left was found to be inaccurate. This discrepancy may be attributed to the median parameter of the Bennett angle on the virtual articulator, potentially leading to insufficiently accurate laterotrusion guidance in the final restoration.

The framework for the final prosthesis was designed and milled with CORiTEC 350i series (imes‐icore GmbH, Germany) and Kera Titanium Disc 98 mm (Eisenbacher Dentalwaren ED GmbH, Germany), ensuring precision and a perfect fit over the implants.

Monolithic zirconia crowns were chosen for their esthetic appeal and strength. These crowns were fabricated using Ceramill Zolid Multilayer A2 Disc (Amann Girrbach AG, Austria), providing a natural translucency that mimicked the appearance of natural teeth [23]. Crea.lign Gum (Bredent) was used to create lifelike gingival prosthetics that blended seamlessly with the patient′s natural gum tissue. All the crowns were carefully cemented extraorally after ensuring passive fitting with the titanium framework, using ZPrime Plus and Duolink Universal (BISCO Inc., United States) (Figure 10c). After that, the prosthesis was seated intraorally directly on the multiunit abutment. The patient′s occlusion was carefully checked after the final prosthesis was seated, including static occlusion and dynamic movement [21, 22]. Some strong contact points were ground down, but not too much, due to the accuracy of the digital system (Figure 10d). The esthetics were also evaluated, focusing on the patient′s facial features and smile line.

**Figure 11:**
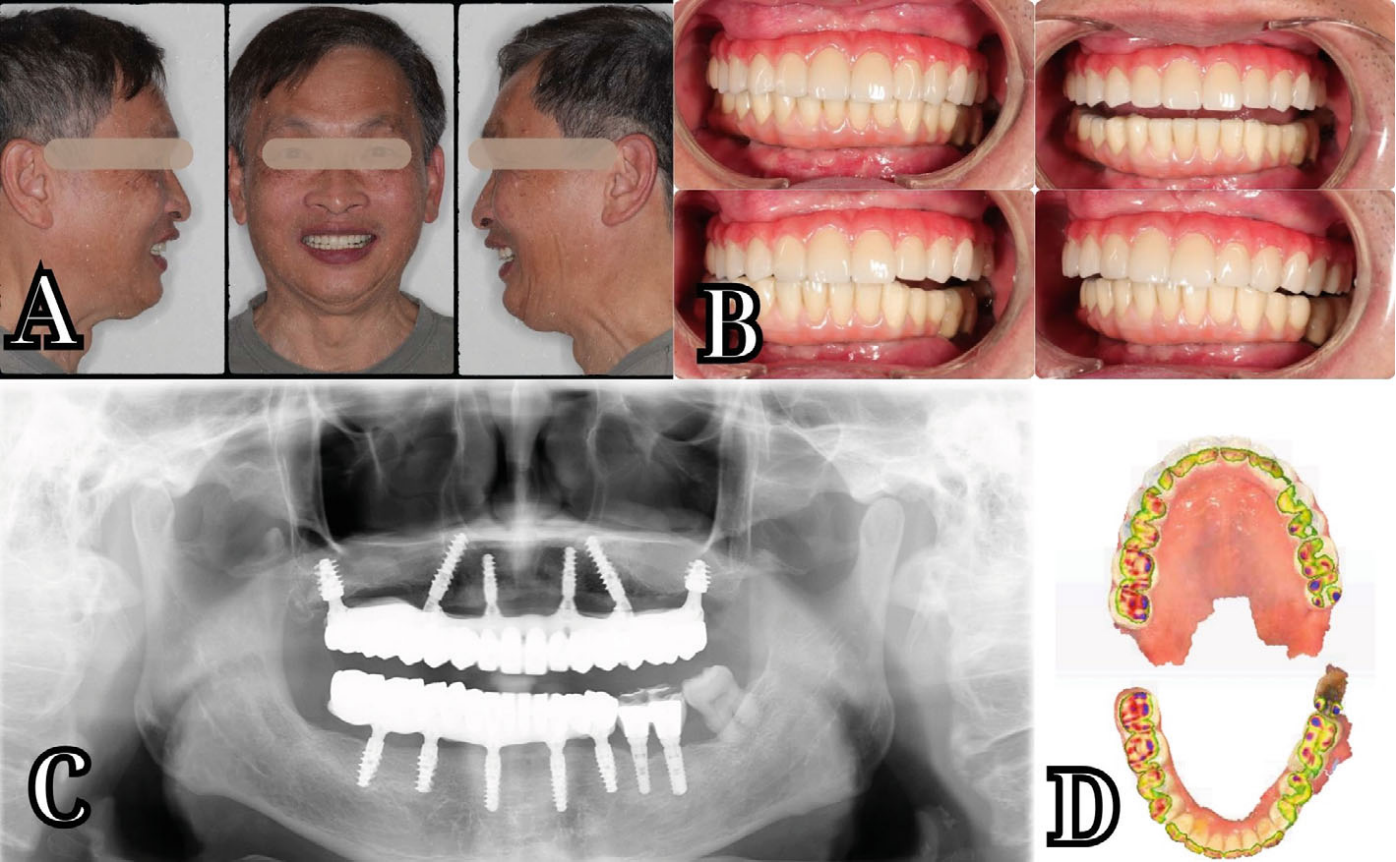
Outcomes. (a) Extraoral photo set in frontal and lateral views. (b) Intraoral photo of static and dynamic occlusion. (c) Panoramic x‐ray confirming the passive fit of the titanium framework and multiunit abutment. (d) Intraoral scan after 6 months.

A final set of intraoral scans and a panoramic x‐ray (Figure 11c) were performed to check the fit and positioning of the prosthesis, confirming that the definitive prosthesis was seated as planned and that the occlusal contacts were even and balanced. Occlusal evaluation was performed using color occlusal paper, Zebris dynamic recordings, and intraoral scans. These complementary assessments demonstrated balanced occlusal contacts in static and dynamic movements, confirming functional stability and reproducibility.

The patient reported excellent results following the delivery of the definitive prosthesis. He experienced improved function, especially in chewing, with no discomfort or sensitivity. Esthetic outcomes were highly satisfactory, with the patient expressing pleasure in both the appearance and functionality of the new restoration (Figure 11a). The patient felt more confident socially and was particularly pleased with the enhanced facial esthetics, which were more youthful due to the restoration of lost vertical dimension.

Follow‐up imaging after 6 months, including a panoramic x‐ray and intraoral scans (Figure 11d), confirmed the stability and precision of the final implant placement and prosthetic fit. There was no evidence of implant failure. The patient was provided with detailed instructions on the maintenance of the prosthesis, including oral hygiene recommendations, regular follow‐up appointments, and advice on avoiding trauma to the prosthetic teeth. Periodic check‐ups were scheduled to monitor implant health and to ensure that the prosthesis continued to perform optimally. He did not experience any adverse events following the placement of the definitive prosthesis. There were no signs of infection, implant failure, or mechanical failure of the prosthesis during the follow‐up period.

## 3. Discussion

This case highlights the benefits of integrating advanced digital workflows into complex implant rehabilitation. The combination of intraoral and facial scans, Exocad virtual planning, stackable guides, the PIC system, and Zebris kinematic records enabled precise implant placement and individualized occlusal design.

The digital workflow was based on four key systems selected for their specific advantages. Exocad was chosen because it allows seamless integration of different data sources (intraoral, facial, and mandibular records) and provides advanced virtual articulator functions, features not available in many other CAD platforms. 3Shape was selected for intraoral scanning and surgical guide design due to its proven accuracy, user‐friendly interface, and strong compatibility with other software, which offered greater clinical reliability than alternative scanning systems. The PIC system was used instead of conventional impression methods or photogrammetry alternatives because it provides highly precise 3D implant position transfer, reducing the risk of misfit and ensuring passive fit of the prosthesis. Finally, the Zebris JMA Optic system was preferred over traditional semiadjustable articulators or average‐value virtual articulators, as it records patient‐specific condylar guidance and functional mandibular movements, enabling individualized occlusal adjustment and enhancing long‐term stability.

Together, these systems were selected because they offered the highest combination of accuracy, compatibility, and clinical efficiency, making them more suitable than alternative approaches for achieving predictable outcomes in complex full‐arch rehabilitation.

Compared with previous reports on digital full‐arch rehabilitation that focused on static protocols, this case demonstrates the added value of incorporating mandibular kinematics. By recording individualized condylar guidance and excursive movements, occlusion was harmonized with the patient′s true functional dynamics. This reduces the risk of occlusal interferences, parafunctional contacts, and uneven load distribution, which can otherwise compromise implant longevity.

From a clinical perspective, the main advantages are twofold. First, implant longevity: Because implants lack a periodontal ligament, they are more vulnerable to overload. Dynamic occlusal adjustment based on condylar pathways distributes forces more evenly, lowering the risk of biomechanical complications such as screw loosening or implant failure. Second, occlusal stability: Balanced functional guidance prevents uneven wear and reduces the need for repeated adjustments, ensuring long‐term comfort and prosthetic stability.

### 3.1. Limitations

Nevertheless, limitations exist. The workflow requires integration of multiple datasets, advanced software, and specialized equipment, which increase cost and complexity. The approach also requires a learning curve for both clinicians and technicians. Finally, reproducibility across different settings remains to be validated through larger case series and long‐term follow‐up studies.

### 3.2. Key Takeaways

Digital workflows, when combined with conventional implant techniques, offer a highly precise and individualized approach to full‐mouth rehabilitation. Incorporating mandibular movement data into the planning and fabrication phases improves both the functionality and comfort of the final prosthesis, leading to better patient outcomes. The success of this case demonstrates that digital and conventional methodologies, when integrated properly, provide a powerful toolset for clinicians to achieve highly accurate and successful implant restorations.

## 4. Conclusion

This case demonstrates that a complete digital workflow incorporating multiple digital tools can provide a highly accurate, compatible, and clinically efficient solution for full‐arch implant rehabilitation. The integration of mandibular movement records enhanced functional predictability, resulting in a stable, esthetic, and functional prosthesis. While advanced equipment and expertise are required, this approach highlights the clinical value of digital technology in improving precision and long‐term outcomes. Future studies with larger cohorts and extended follow‐up are necessary to validate these findings and expand their applicability in daily practice.

## Ethics Statement

This case report was conducted in accordance with the ethical principles of the Declaration of Helsinki. Formal Institutional Review Board (IRB) approval was not required for a single‐patient case report. Written informed consent was obtained from the patient for both the treatment procedure and the publication of the case details and accompanying images. All identifying information has been omitted to protect patient confidentiality.

## Consent

Informed consent was obtained from the patient for the use of their medical data, images, and case details in this report. The patient agreed to have their anonymized information included in the case report for educational and research purposes.

## Patient Perspective

The patient expressed high levels of satisfaction with both the functional and esthetic outcomes of the full‐arch rehabilitation. He reported being able to chew comfortably and more confidently, and he was particularly pleased with the improved appearance of his smile. The patient noted that he felt a significant boost in his self‐esteem and was eager to show off his new smile.

## Conflicts of Interest

The authors declare no conflicts of interest.

## Author Contributions

K.L.N. contributed to conceptualization, methodology, validation, formal analysis, investigation, resources, writing—review and editing, supervision, project administration, and funding acquisition. Q.K.C. contributed to methodology, software, formal analysis, resources, data curation, writing—original draft, and visualization. D.T.D. contributed to software, investigation, resources, and writing—original draft. T.T.N. contributed to investigation, resources, and writing—original draft.

## Funding

No funding was received for this manuscript.

## Data Availability

The data that support the findings of this study are available from the corresponding author upon reasonable request.
